# Easter Day 1917: The Murder of Non-Combatants

**Published:** 1917-04-07

**Authors:** 


### The Hospital

The Workers' Newspaper of Administrative Medicine and Institutional Life, Administration, National Insurance and Health.

No. 1609, Vol. LXII. SATURDAY, APRIL 7, 1917. PRICE ONE PENNY

230058

### EASTER DAY 1917: THE MURDER OF NON-COMBATANTS.

Easter Day 1917 will be the third Easter under the increasing inhumanities of the Germans and their allies in the Great War. This war has now become, so far as the enemy is concerned, a war of frightfulness, to use the German term, that is one which has produced untold horrors, outrages, murders, slaveries, starvations, bestialities, and abominations so unnamable as to make their perpetrators as a nation and people worse, in fact, in conduct and action than anything the animal kingdom in its wildest and most barbarous condition has produced. Within a week of the commencement of the war by Germany we published an article entitled "War and Insanity." In the course of that article we enforced the truth that the war in its origin rested upon moral degeneracy and absence of sound judgment. Its authors had persuaded themselves that the money they had accumulated, the armaments and munitions of war which they had created, and the control of enormous armies they had drilled to the use of these implements of destruction, would prove irresistible.

A lunatic believes that he is irresistible, and not infrequently believes he is the Deity himself. We ventured to declare that it must be plain to all thoughtful minds that the nations of the earth should combine quickly to stamp out this war, by placing the authors of it in safe confinement, out of mischief. We declared they could never be permitted to control, to rule, and to enslave the world. At the outset of the war three Great Powers and several small ones had had war declared against them by the madmen who were responsible for the Armageddon. One great European Power, Italy, stood neutral, and there was also the United States of America, which had no direct interest in the causes which underlay the original troubles.

That was the position in August 1914, which we put shortly thus: "A relatively few madmen, fired by inordinate greed and ambitions, have for their own purposes converted Europe into a huge battlefield. When the civilised nations of the earth realise this to be so, it seems a certainty that, every country possessed of a sane Government will spontaneously come forward and join hands with other resisting peoples, with the view of stopping the demented and guilty beings responsible. Civilisation demands the unity of civilised nations to guarantee the sober government of the world and the defeat of insanity." Such was our forecast and conclusion, resting upon a study of the German nature, as revealed to us from personal investigations, made throughout Germany before the war.

Since then three Easters have come, without Christ's teaching finding the faintest expression through the "cultured" German and his actions in wartime. On the contrary, the Germans have gone from bad to worse, until Easter Day 1917 may be celebrated by the wholesale murder of non-combatants on the high seas by command of the rulers and controllers of Germany and her allies, whom we ventured to describe as lunatics, who believed that they were irresistible, but were really moral degenerates out to control, endeavouring to rule and to enslave the world. We have already in very imperfect language enumerated some of the principal horrors and abominations of the enemy. They have been such as to compel the Italian nation and its rulers to join hands with the Allies, and as we go to press a cable message has come from the United States containing the decision of President Wilson, speaking for a united nation. The President denominates our enemies' proceedings as "the wanton and wholesale destruction of the lives of non-combatants—men, women, and children—engaged in pursuits which have always, even in the darkest periods of modern history, been deemed innocent and legitimate." President Wilson describes Germany's action as warfare on mankind. "Property can be paid for; the lives of peaceful and innocent people cannot be. The present German warfare against commerce is warfare against mankind. It is a war against all nations. The challenge is to all mankind, and our motive will not be revenge, but only a vindication of right, of human right." The President rejects the path of submission, and declines to suffer the most sacred rights of the American nation and people to be ignored and violated. He advises Congress to declare the recent course of the Imperial German Government to be in fact, nothing less than war against the Government and people of the United States. Mr. Wilson declares Right is more precious than Peace. He invites his countrymen to dedicate "their lives, their fortunes—everything they are, everything they have —with the pride of those who know the day has come when America is privileged to spend her blood and might, for the principles that gave her birth, and the happiness and peace which she has treasured. God helping her, she can do no other."

Our forecast is well on its way to complete fulfilment. Easter Day 1917 finds the civilised world united in upholding "the universal dominion of right by such a concert of free peoples as will bring peace and safety to all nations and make the world itself at last free." The Motherland thanks God and takes courage at the union of all her children under such a flag in such a cause.

When closer strand shall lean to strand
And meet, beneath saluting flags,
The eagle of our mountain crags,
The lion of our mother-land.

